# Immunomodulatory Effects of SPHK1 and Its Interaction with TFAP2A in Yellow Drum (*Nibea albiflora*)

**DOI:** 10.3390/ijms252413641

**Published:** 2024-12-20

**Authors:** Yu Cui, Shuai Luo, Baolan Wu, Qiaoying Li, Fang Han, Zhiyong Wang

**Affiliations:** 1 State Key Laboratory of Mariculture Breeding, Key Laboratory of Healthy Mariculture for the East China Sea, Fisheries College, Jimei University, Xiamen 361021, China; 18687492995@163.com (Y.C.); law_seo@163.com (S.L.); wbl5160691422@163.com (B.W.); 17601168505@163.com (Q.L.); 2 Laboratory for Marine Fisheries Science and Food Production Processes, Qingdao National Laboratory for Marine Science and Technology, Qingdao 266237, China

**Keywords:** *sphk1*, *Nibea albiflora*, sphingosine kinase, *Vibrio harveyi*, RNA sequencing, immune regulation

## Abstract

Sphingosine kinases (SPHKs) are essential enzymes that catalyze the phosphorylation of sphingosine to produce sphingosine-1-phosphate (S1P), which plays pivotal roles in inflammation and immune regulation. In this study, genome-wide association analysis (GWAS) identified the *Ydsphk1* gene as closely associated with the resistance of yellow drum (*Nibea albiflora*) to *Vibrio harveyi*. Structural prediction showed that YDSPHK1 contains a typical diacylglycerol kinase catalytic (DAGKc) domain (154–291 aa). By constructing and transfecting *Ydsphk1* expression plasmids into yellow drum kidney cells, we found that YDSPHK1 is localized in the cytoplasm. Subsequent RNA-Seq analysis of an overexpression plasmid identified 25 differentially expressed genes (DEGs), including 13 upregulated and 12 downregulated. Notably, *nsun5* and *hsp90aa1* were significantly upregulated, while *Nfkbia* and *hmox1* were downregulated. Promoter analysis indicated that the core regulatory regions of *Ydsphk1* are located between −1931~−1679 bp and −419~+92 bp, with two predicted TFAP2A binding sites in the −419~+92 bp region. Further studies demonstrated that varying concentrations of TFAP2A significantly reduced *Ydsphk1* promoter activity. These findings underscore the pivotal role of *Ydsphk1* in regulating immune responses in yellow drum, particularly through its impact on key immune-related genes and pathways such as NF-κB signaling and ferroptosis. The identification of *Ydsphk1* as a mediator of immune regulation provides valuable insights into the molecular mechanisms of immune defense and highlights its potential as a target for enhancing pathogen resistance in aquaculture practices. This study lays a strong foundation for future research aimed at developing innovative strategies for disease management in aquaculture species.

## 1. Introduction

Aquaculture, particularly for species like yellow drum (*Nibea albiflora*), is vital to China’s southeastern coastal economy. However, bacterial infections, especially from *Vibrio harveyi*, pose a significant threat, leading to high mortality rates and substantial economic losses [1,2,3,4,5]. Effective strategies to combat such infections depend on a thorough understanding of the molecular mechanisms governing immune responses in fish.

Sphingosine kinases (SPHKs) are key enzymes in sphingolipid metabolism, catalyzing the conversion of sphingosine to sphingosine-1-phosphate (S1P), a bioactive lipid involved in numerous cellular processes, including cell proliferation, migration, apoptosis, and immune regulation [6,7] Intracellularly, S1P interacts with TRAF2, activating the NF-κB pathway and regulating immune responses and inflammation [6,8,9,10,11,12,13,14]. Extracellularly, S1P functions as a ligand that binds to specific G-protein coupled receptors (GPCRs) such as EDG receptors, triggering downstream signaling pathways that promote cell survival and vascular integrity [15,16,17]. The balance between S1P, sphingosine, and ceramide is tightly controlled by sphingosine kinases, with SPHK1 playing a central role in regulating these processes [18].

In mammals, SPHK1 has been extensively studied for its role in cancer progression, particularly in promoting invasion and metastasis through the epithelial–mesenchymal transition (EMT) pathway [19,20]. SPHK1 promotes inflammation by activating the NF-κB signaling pathway, which leads to the phosphorylation of JNK (c-Jun N-terminal kinase) and the activation of downstream JNK-Jun and JNK-ATF3 signaling cascades. SphK1 activation is linked to NF-κB-mediated inflammatory responses [21,22]. Knockout studies in zebrafish have demonstrated that loss of sphk1 leads to abnormal development and heightened immune responses, underscoring its evolutionary conservation and importance in immune function [23]. Studies have demonstrated that in yellow drum, the NF-κB signaling pathway is modulated by multiple immune genes, triggering an effective immune response [24,25]. In fish, the role of sphingosine kinase 1 (sphk1) in immune responses remains less understood, but recent studies in yellow drum have highlighted its significance. Genome-wide association studies (GWASs) identified *Ydsphk1* as a gene associated with resistance to *V. harveyi* infections [4]. This gene exhibits high expression in immune organs, particularly the liver, where its expression can increase up to 39-fold following bacterial infection [4]. Such dramatic upregulation suggests that YDSPHK1 plays a crucial role in modulating the immune response, potentially regulating key inflammatory and stress pathways like NF-κB and ferroptosis [26,27].

To better understand the molecular mechanisms of *Ydsphk1* in the immune system of yellow drum, this study investigates its protein structure, subcellular localization, and transcriptional regulation. Specifically, we examine the role of the transcription factor TFAP2A, which is predicted to negatively regulate *Ydsphk1* promoter activity through two binding sites located in the core promoter region. Given that TFAP2A has been implicated in oncogenic and immune regulatory pathways in other species [28], understanding its interaction with YDSPHK1 could provide novel insights into immune regulation in fish. This research aims to enhance our understanding of immune mechanisms in yellow drum, with potential implications for improving disease resistance in aquaculture.

## 2. Results

### 2.1. Structure and Subcellular Localization Analysis of Ydsphk1

**Figure 1:**
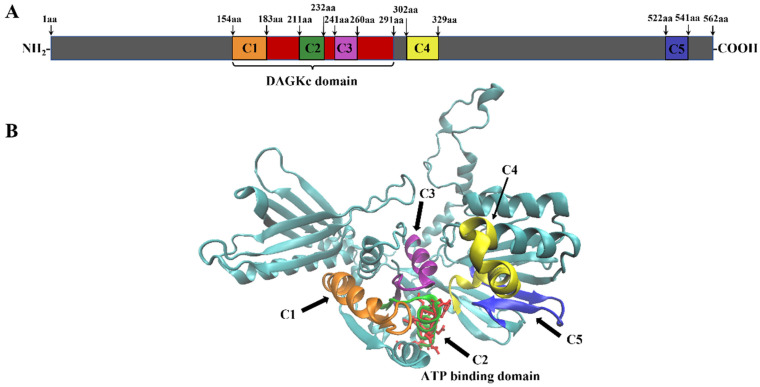
Protein structures of YDSPHK1 in *N. albiflora*. (**A**) The position of five conserved domains (C1–C5) and the DAGKc domain within the sequence and (**B**) the predicted tertiary structure. Brown represents conserved domain C1, green represents C2, purple represents C3, yellow represents C4, blue represents C5, and the DAGKc domain is highlighted in red.

**Figure 2:**
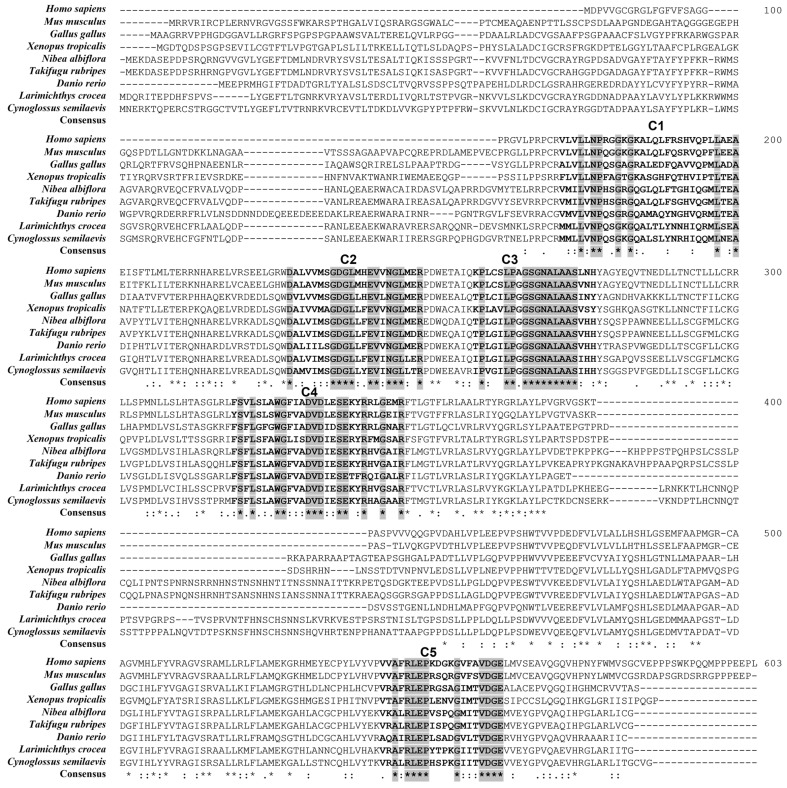
Multiple sequence alignment of YDSPHK1 in *N. albiflora*. The five conserved domains (C1–C5) are shown in bold black letters, and conserved residues are highlighted in gray. (*) indicates fully conserved residues, (:) represents residues with strongly similar properties, and (.) indicates residues with weakly similar properties.

SMART protein domain analysis revealed that YDSPHK1 contains a typical diacylglycerol kinase catalytic (DAGKc) domain (154–291 aa), which is essential for its catalytic activity (Figure 1A). The predicted tertiary structure of YDSPHK1 is primarily composed of alpha-helices, consistent with its role as a sphingosine kinase (Figure 1B). Multiple sequence alignment across various species, including fish, mammals, birds, and amphibians, showed that five conserved domains (C1–C5) are well-preserved, indicating a high degree of functional conservation (Figure 2).

**Figure 3:**
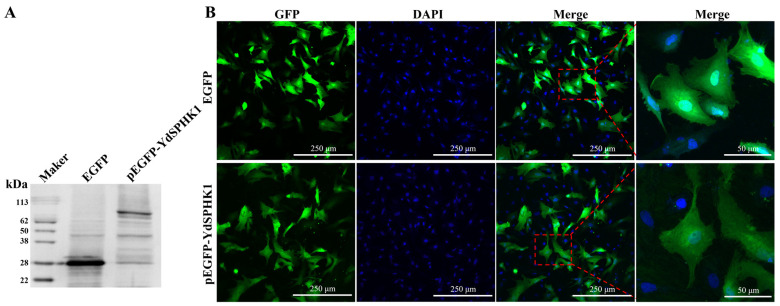
Western blot analysis (**A**) and subcellular localization (**B**) of YDSPHK1 in *N. albiflora* kidney cells. (**A**) Western blot showing the expression of pEGFP-N1 (lane 1) and pEGFP-YDSPHK1 fusion protein (lane 2). (**B**) Subcellular localization of YDSPHK1 observed via GFP fluorescence. pEGFP-N1 serves as the control, while pEGFP-YDSPHK1 is expressed in both the cytoplasm and the nucleus.

Western blot analysis confirmed the expected molecular weights of the fusion proteins pEGFP-N1 (~26 kDa) and pEGFP-YDSPHK1 (~88 kDa), aligning with predicted sizes (Figure 3A). A subcellular localization study indicated that YDSPHK1 is primarily distributed in the cytoplasm of *N. albiflora* kidney cells, consistent with its function in sphingolipid metabolism (Figure 3B).

### 2.2. RNA-Seq Analysis of Ydsphk1 Overexpression Effects

**Figure 4:**
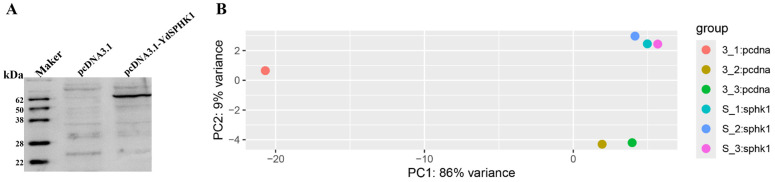
Western blot analysis of pcDNA3.1(-) Myc-His B-YDSPHK1 and principal component analysis (PCA) of six samples. (**A**) Western blot showing the expression of the pcDNA3.1(-) Myc-His B-YDSPHK1 fusion protein. (**B**) Principal component analysis (PCA) of six samples, illustrating the distinct clustering of experimental and control groups.

To assess the functional role of YDSPHK1 in immune responses, RNA-Seq was performed on kidney cells overexpressing YDSPHK1. Western blot analysis confirmed the successful expression of YDSPHK1 (~64 kDa) in the transfected cells (Figure 4A). A total of 41.69 Gb of raw RNA-Seq data were obtained, with 40.05 Gb of clean data retained after quality control and a 100% mapping rate to the *N. albiflora* genome (Table 1). Transcript assembly identified 23,345 transcripts (Supplementary Table S1), and 17,051 genes were annotated (Supplementary Table S2).

**Figure 5:**
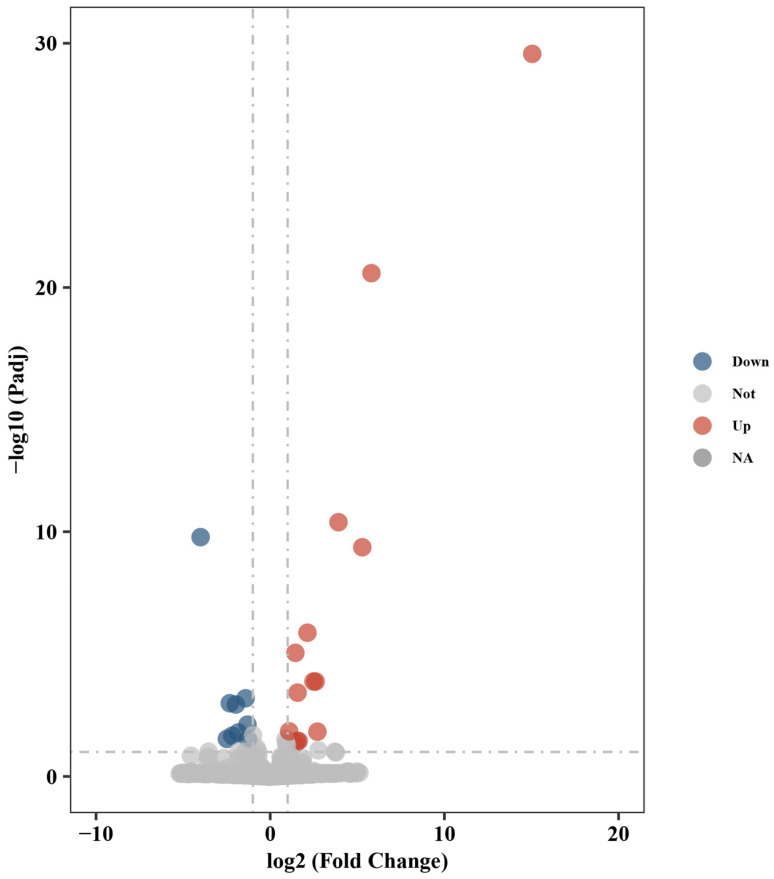
The volcano plot of differently expressed genes. The plot illustrates the distribution of upregulated and downregulated genes, with significant differentially expressed genes highlighted.

**Figure 6:**
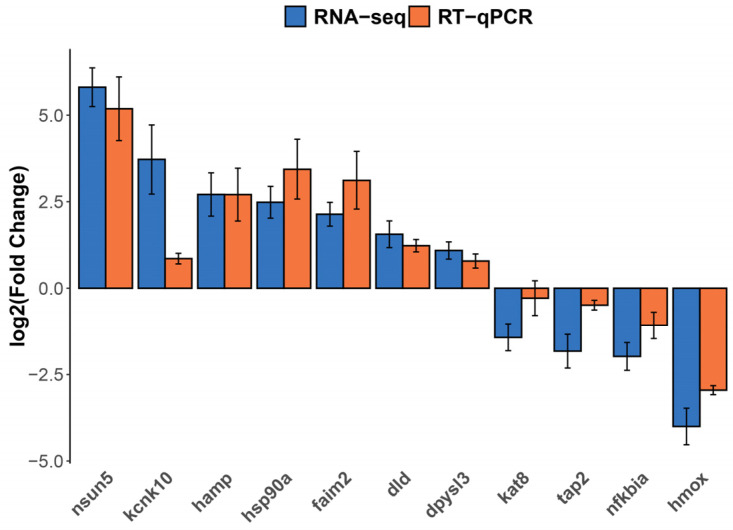
Gene expression patterns of RNA-Seq and RT-qPCR. The *x*-axis represents the log2(fold change), where positive values indicate upregulated genes, and negative values indicate downregulated genes. The *y*-axis represents the differential genes analyzed in the study.

Principal component analysis (PCA) indicated clear clustering between the control and experimental groups (Figure 4B); it indicated that the control group sample 3-1 was an outlier. Therefore, this sample was excluded, and confirming distinct expression profiles was subsequently performed with the remaining data. Differential gene expression analysis revealed 25 differentially expressed genes (DEGs), including 13 upregulated and 12 downregulated genes (Table 2, Figure 5). RT-qPCR confirmed expression levels consistent with transcriptome data (Figure 6).

**Figure 7:**
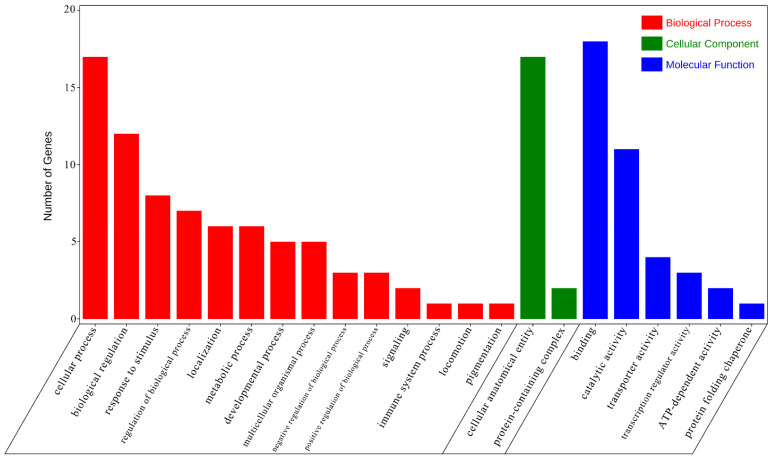
GO function annotation of differentially expressed genes (DEGs). The figure shows the Gene Ontology (GO) classification of differentially expressed genes, categorized into biological processes, cellular components, and molecular functions.

**Figure 8:**
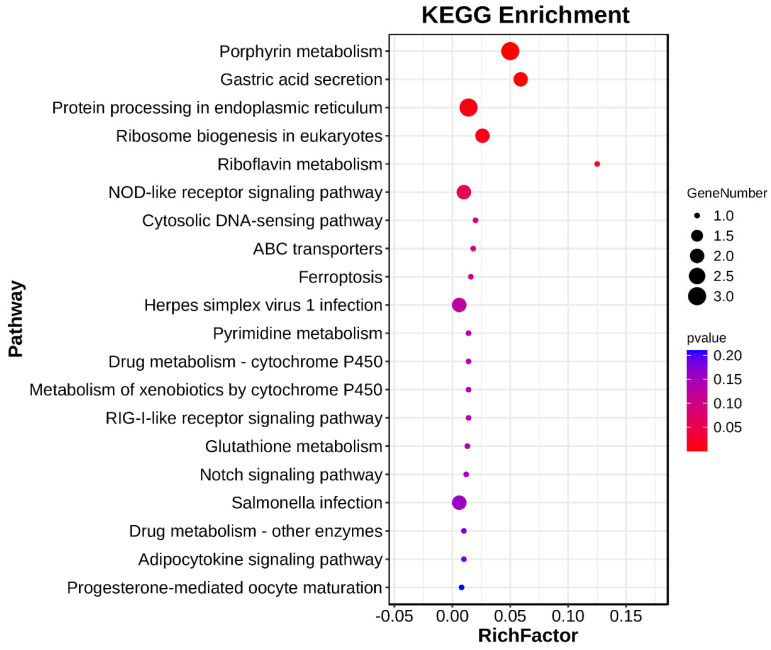
KEGG functional enrichment of differently expressed genes. The figure displays the functional enrichment of differentially expressed genes, highlighting significantly enriched pathways identified through GO (Gene Ontology) and KEGG (Kyoto Encyclopedia of Genes and Genomes) analyses. These pathways are categorized based on their involvement in biological processes, cellular components, molecular functions, and metabolic or immune-related pathways.

GO enrichment analysis indicated that several differentially expressed genes (DEGs) are implicated in immune responses. Within the Biological Process (BP) category, these DEGs were predominantly associated with cellular processes, response to stimulus, and immune system processes, including genes such as *dpysl3*, *nfkbia*, *dld*, *hsp90aa1*, *hmox*, *tap2*, *faim2*, and *kat8*. In the Cellular Component (CC) category, the enriched terms pertained to cellular anatomical entities and protein-containing complexes. For the Molecular Function (MF) category, the DEGs were primarily linked to binding and catalytic activities (Figure 7). Additionally, KEGG pathway analysis revealed significant enrichment in various signaling pathways, such as porphyrin metabolism, NOD-like receptor signaling pathway, Salmonella infection, ferroptosis, Notch signaling pathway, and herpes simplex virus 1 infection, involving *hsp90aa1* and *nfkbia* (Figure 8).

### 2.3. Promoter Activity of Ydsphk1

**Figure 9:**
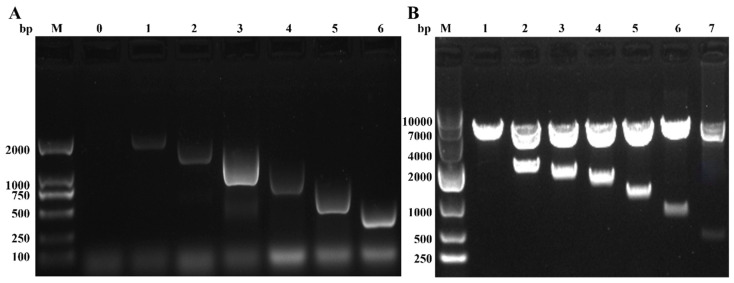
Cloning of six *Ydsphk1* promoter fragments and confirmation of recombinant vector by double digestion. (**A**) Schematic representation of the six cloned *Ydsphk1* promoter fragments, each containing varying lengths of the promoter region. (**B**) Agarose gel electrophoresis showing the results of the double digestion of the recombinant vectors, confirming successful cloning of the *Ydsphk1* promoter fragments into the vector. Lanes 1–7 represent the full-length promoter and different truncated fragments (from pGL3-SPHK1-P1 to P6), while lane M represents the marker.

**Figure 10:**
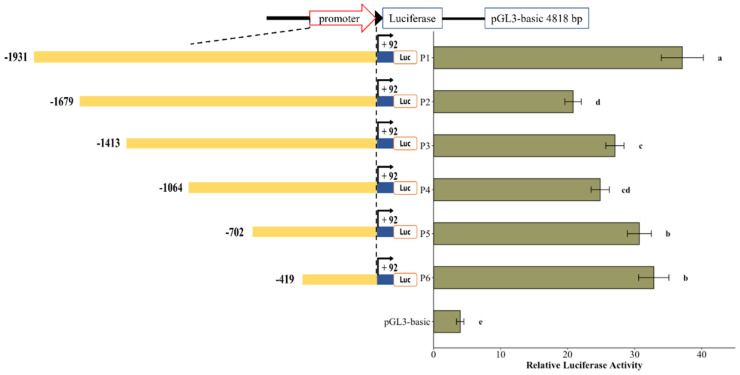
Promoter activity of different *Ydsphk1* fragments. The 5′ deletion constructs of promoter are shown on the left yellow bars, with the coding exon and luciferase coding frames represented by closed boxes. The sequence is numbered relative to the first base of the start codon (ATG). The promoter activity of each construct is displayed on the right, with values representing normalized luciferase activity (Firefly luciferase/Renilla luciferase). Letters indicate significant differences (*p* < 0.05) among the different deletion constructs.

The activity of various *Ydsphk1* promoter deletion fragments was measured using a dual-luciferase reporter assay (Figure 9). The deletion constructs revealed that the −1931~−1679 and −419~+92 regions are critical for the positive transcriptional regulation of *Ydsphk1*, while the −1679~−1413 region contains potential negative regulatory elements. The highest promoter activity was observed for the longest construct, pGL3-SPHK1-P1, and the lowest for pGL3-SPHK1-P2 (Figure 10).

### 2.4. Regulation of Ydsphk1 by TFAP2A

**Figure 11:**
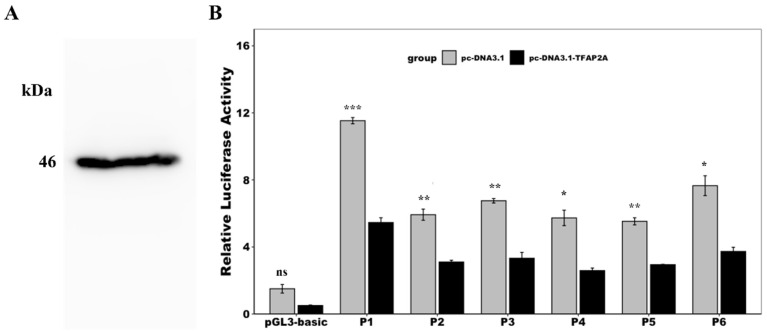
Effect of TFAP2A overexpression on the activity of the *Ydsphk1* promoter in yellow drum. (**A**) Western blot detection of TFAP2A recombinant protein (~46 kDa). (**B**) Effect of TFAP2A overexpression on the activity of the *Ydsphk1* promoter. The asterisk (*) indicates a significant difference in the activity of the various truncated promoter fragments compared to the control group (pGL3-basic) (* *p* < 0.05; ** *p* < 0.01; *** *p* < 0.001; ns: not significant).

Two potential TFAP2A binding sites were identified in the core promoter region (−419~+92) of *Ydsphk1*. To investigate the regulatory role of TFAP2A on *Ydsphk1* expression, we transfected 293T cells with TFAP2A recombinant plasmids and co-transfected them with various deletion fragments of the *Ydsphk1* promoter. Western blot analysis confirmed the successful expression of TFAP2A (~46 kDa) in 293T cells (Figure 11A).

**Figure 12:**
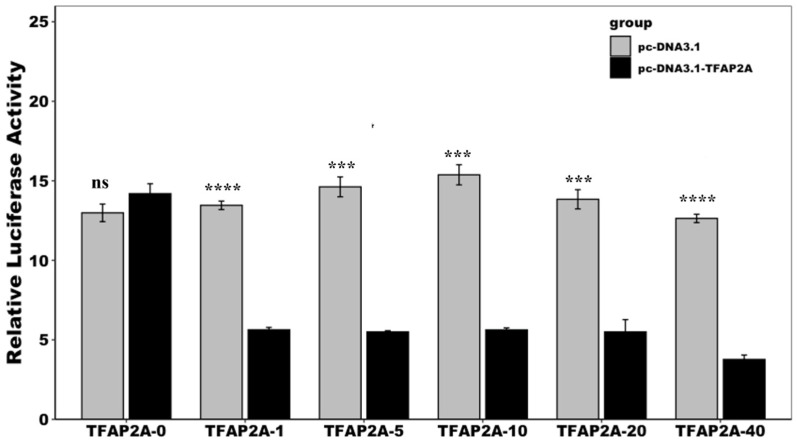
The effect of different doses of TFAP2A overexpression on the activity of the *Ydsphk1* promoter. The figure illustrates the impact of varying doses of TFAP2A overexpression on *Ydsphk1* promoter activity. Promoter activity was measured as normalized luciferase activity (Firefly luciferase/Renilla luciferase), and significant differences between doses were assessed. (*** *p* < 0.001; **** *p* < 0.0001; ns: not significant).

A dual-luciferase reporter assay demonstrated that TFAP2A significantly decreased the activity of the *Ydsphk1* promoter under all tested conditions (Figure 12). Although different concentrations of TFAP2A plasmids were introduced (ranging from 10 ng to 200 ng), no clear dose-dependent effect was observed. However, at the highest concentration (200 ng), promoter activity was reduced by approximately 50% compared to the control group (Figure 12). This suggests that while TFAP2A serves as a negative regulator of *Ydsphk1* transcription, its effect may plateau at higher concentrations, indicating potential saturation of TFAP2A binding sites.

These findings suggest that TFAP2A can repress *Ydsphk1* promoter activity; however, the absence of a clear dose–response curve suggests that other factors may influence the degree of repression at higher concentrations.

## 3. Discussion

This study has identified *Ydsphk1* as a key regulator in the immune response of *N. albiflora* (yellow drum) against *V. harveyi* infection, providing valuable insights into immune regulation in fish. Transcriptome analysis revealed 13 significantly upregulated genes and 12 downregulated genes after YDSPHK1 overexpression. GO and KEGG enrichment analyses indicated that many differentially expressed genes (DEGs) were involved in metabolic processes, apoptosis, and immune responses, highlighting the multifaceted role of YDSPHK1 in orchestrating the immune response.

Among the upregulated genes, *Nsun5* (RNA methyltransferase 5) is known for its role in mRNA methylation and its association with tumor invasion and metastasis in humans. In gliomas, Nsun5 modifies cytoplasmic RNA through 5-methylcytosine (m5C) modification, which is then oxidized by TET2 into 5-hydroxymethylcytosine (5hmC), leading to RNA degradation and inhibition of tumor growth [29,30,31]. This upregulation suggests that *nsun5* might also play a role in regulating cell death or immune responses in *N. albiflora*, possibly influencing immune homeostasis during infection. Similarly, *Hsp90aa1*, a molecular chaperone involved in protein folding, was upregulated. In humans, Hsp90aa1 is associated with poor prognosis in cancers such as leukemia and breast cancer [32,33,34,35]. Its upregulation during bacterial infection in fish may indicate a role in stress responses and cellular protection, possibly by stabilizing key immune-related proteins.

Other upregulated genes, such as *dld* (dihydrolipoamide dehydrogenase) [36,37], *faim2* (Fas apoptotic inhibitory molecule 2) [38], and *dpysl3* (dihydropyrimidinase-like 3) [39], have been linked to poor cancer outcomes in humans. Their upregulation in yellow drum suggests that they may also contribute to regulating immune responses by modulating apoptosis and cell survival. These findings support the hypothesis that *Ydsphk1* not only regulates immune pathways but also modulates the balance between cell survival and programmed cell death, which is critical during infection.

In the downregulated group, *nfkbia* (IκBα) plays a crucial role in regulating the NF-κB signaling pathway, a key pathway involved in immune responses [40]. The downregulation of *nfkbia* suggests that the NF-κB pathway is activated in response to *V. harveyi* infection, enhancing the immune defense mechanism in *N. albiflora.* This activation is consistent with previous studies showing that NF-κB is a critical regulator of inflammation and immune responses across various species [23,41,42]. Additionally, *kat8* (lysine acetyltransferase 8), which was also downregulated, has been shown to negatively regulate antiviral immune responses. Studies in mice have demonstrated that kat8 knockdown enhances resistance to infection [43], suggesting that decreased kat8 expression in yellow drum may similarly boost antiviral defenses during bacterial infection.

Ferroptosis is a form of regulated cell death linked to iron metabolism, believed to be a mechanism by which cells respond to infection and stress [44,45,46]. Growing evidence indicates that pathogens can induce ferroptosis in host cells to enhance their pathogenicity and facilitate their spread [47]. For instance, bacteria such as *Pseudomonas aeruginosa*, *Mycobacterium tuberculosis*, and *Salmonella* have been shown to trigger ferroptosis in host cells. In fish, ferroptosis and iron metabolism play a role in the immune response against *Pseudomonas plecoglossicida* in orange-spotted groupers [48]. Additionally, *Escherichia coli* can induce ferroptosis in the red blood cells of grass carp [49].

In this study, we observed that ferroptosis-related genes, such as *hamp* [50] and *hmox1* [51], were significantly downregulated in response to infection. The downregulation of these genes may represent a protective mechanism, potentially limiting excessive cell death during immune responses to infection. This suggests that ferroptosis, although typically associated with stress-induced cell death, may also be carefully regulated to avoid excessive tissue damage while combating infection.

The upregulation of *Ydsphk1* is particularly noteworthy, as previous studies in humans have demonstrated that SPHK1 plays a key role in regulating immune responses, particularly through its interaction with PD-L1 and other immune checkpoint molecules [6,52,53,54]. SPHK1 inhibitors, such as RB-005 and SK1-I, have been developed for the treatment of inflammatory diseases and cancers [55,56]. Building on these findings, we hypothesize that *Ydsphk1* in *N. albiflora* may not directly contribute to susceptibility to *V. harveyi* but instead serves as a mediator of immune responses, particularly through the regulation of programmed cell death and apoptosis. Targeting *Ydsphk1* could potentially mitigate excessive inflammation and reduce mortality in fish infected with *V. harveyi*, making it a promising target for disease management in aquaculture.

A novel finding of this study is the regulation of *Ydsphk1* by the transcription factor *TFAP2A*. Two potential *TFAP2A* binding sites were identified within the core promoter region of *Ydsphk1*, and functional assays confirmed that *TFAP2A* acts as a negative regulator of *Ydsphk1* transcription. Interestingly, *TFAP2A* has been shown to have dual roles in other species, acting as both a tumor suppressor and an oncogene depending on the cellular context [28,54,57]. Our results indicate that *TFAP2A* significantly reduces *Ydsphk1* promoter activity, suggesting that this regulatory mechanism may serve as a critical checkpoint in immune responses, modulating *Ydsphk1* expression during the early stages of infection. However, the absence of a clear dose-dependent relationship between *TFAP2A* concentration and promoter activity reduction suggests that additional regulatory factors may influence this transcriptional network.

Most differentially expressed genes, including *Ydsphk1*, have been linked to poor cancer prognosis and programmed cell death (apoptosis). We hypothesize that the elevated expression of *Ydsphk1* may suppress *Nfkbia*, thereby activating the NF-κB signaling pathway in response to immune stimuli. Notably, previous studies showed that mortality began at 72 h post-challenge, peaking at 144 h, while *Ydsphk1* expression peaked at 24 h post-challenge. This temporal correlation suggests that *Ydsphk1* upregulation could serve as an early biomarker for *V. harveyi* infection in aquaculture.

Future research should further explore the interactions between TFAP2A-YDSPHK1- NFKBIA and other immune pathways, such as NF-κB signaling and ferroptosis, to comprehensively elucidate its role in immune regulation and identify potential interventions for improving disease resistance in aquaculture species.

In conclusion, our study enhances the understanding of *Ydsphk1* as an important regulator of immune responses in *N. albiflora*. The transcriptional regulation of *Ydsphk1* by TFAP2A adds complexity to the immune regulatory network in fish and provides a potential target for therapeutic interventions. This discovery advances our understanding of transcriptional control in fish immunity and suggests *Ydsphk1* as a potential target for enhancing disease resistance in aquaculture. Future studies should focus on further exploring the molecular mechanisms governing *Ydsphk1* regulation and its interactions with other immune pathways, particularly NF-κB signaling, to fully exploit its potential in aquaculture disease control.

## 4. Materials and Methods

### 4.1. Gene Characterization

The protein sequence of *YDSPHK1* was compared with other species using the NCBI BLAST tool (https://blast.ncbi.nlm.nih.gov/Blast.cgi, accessed on 15 August 2023). Homologous sequences were downloaded and aligned using ClustalW (https://www.ebi.ac.uk/Tools/msa/clustalo/, accessed on 25 August 2023.). The conserved domains were predicted using SMART (http://smart.embl-heidelberg.de, accessed on 25 August 2023). The tertiary structure was modeled using SWISS-MODEL (https://swissmodel.expasy.org/, accessed on 7 October 2023) and tFold (https://drug.ai.tencent.com/console/cn/tfold, accessed on 7 October 2023). The structures were visualized using VMD 1.9.2 (https://www.ks.uiuc.edu/Research/vmd/VMD-1.9.2, accessed on 12 October 2023).

### 4.2. RNA Extraction and cDNA Synthesis

Both the experimental and control groups included three biological replicates each. Total RNA was extracted using the EasyPure^®^ Fast Cell RNA Kit (TransGen Biotech, Beijing, China) according to the manufacturer’s protocol.

The cells were centrifuged at 1000× *g* for 5 min at 4 °C, and the pellet was retained. To lyse the cells, 500 µL of LB50 buffer was added, followed by vortexing to mix thoroughly. After a 1 min incubation at room temperature, the lysate was transferred to a spin column and centrifuged at 13,500× *g* for 30 s. The flow-through was discarded.

The column was washed with 500 µL of WB50 buffer and centrifuged at 13,500× *g* for 30 s, discarding the flow-through. This step was repeated twice to ensure cleanliness. The column was then centrifuged at 13,500× *g* for 2 min to remove residual ethanol.

To elute the RNA, the column was placed in a new 1.5 mL tube, and 50 µL of RNase-free water was added to the center of the membrane. After a 1 min incubation at room temperature, the column was centrifuged to collect the purified RNA.

After determining the RNA concentration, 1 µg of total RNA was used for first-strand cDNA synthesis, which was performed using the GoScript™ Reverse Transcription System (Promega, Madison, WI, USA).

### 4.3. Subcellular Localization of YDSPHK1

The subcellular localization of YDSPHK1 was studied by transfecting the kidney cells of *N. albiflora* with *pEGFP-N1* (empty plasmid) and *pEGFP-YDSPHK1* (recombinant plasmid, Table 3) using the Generation Electroporator (BEX, Tokyo, Japan). The kidney cells of *N. albiflora* used in this study were cultured in our laboratory, have been stably passaged, and are deposited in the China Center for Type Culture Collection with the CCTCC NO:C2023222. Cells were cultured as described by Luo [58]. After 24 h of transfection, Western blot analysis was performed to detect YDSPHK1 expression. Cells were lysed, and protein samples were separated using 12.5% SDS-PAGE and then transferred onto PVDF membranes. The membrane was blocked, incubated with GFP-tag polyclonal antibodies (diluted 1:4000, Proteintech, Rosemont, IL, USA), followed by a secondary antibody, and visualized using the BeyoECL Plus chemiluminescence kit (Beyotime, Shanghai, China). Fluorescence images were captured with the Leica TCS SP8 laser scanning confocal microscope system (Leica, Wetzlar, Germany).

### 4.4. Transcriptome Sequencing and Differentially Expressed Genes (DGEs) Analysis

Kidney cells of *N. albiflora* were cultured in a 12-well cell plate; the average cell yield was about 2.3 × 10^5^, with 6 wells assigned to the experimental group transfected with *pcDNA3.1(-) Myc-His B-YDSPHK1* and 6 wells assigned to the control group transfected with *pcDNA3.1(-) Myc-His B*. Total RNA was extracted from cells in 2 wells and used as a biological sample. Total RNA was extracted using the EasyPure^®^ Fast Cell RNA Kit (TransGen Biotech, Beijing, China).

The RNA quality was assessed with a NanoDrop 2000 spectrophotometer (Thermo Scientific, Waltham, MA, USA), and RNA integrity was confirmed by agarose gel electrophoresis. The RIN (RNA Integrity Number) values of the samples ranged from 7.2 to 10, meeting the requirements for sequencing. Qualified RNA samples were sent to Novogene (Beijing, China) for sequencing on the Illumina NovaSeq 6000 platform.

After obtaining the sequencing data, quality control was conducted using FASTP (0.23.4) to filter out low-quality reads. Clean reads were then aligned to the *N. albiflora* reference genome using STAR [59] with --clip5pNbases 0 5, --clip3pNbases 0 25, --outSJfilterOverhangMin 30 16 16 16, --outSJfilterCountUniqueMin 4 2 2 2, --alignSJoverhangMin 6, --alignIntronMax 500000, --alignMatesGapMax 500000, --outFilterMismatchNoverReadLmax 1.0. The aligned reads were assembled into transcripts using StringTie [60] with -f 0.1, -j 2, -c 4. and gene expression was quantified in TPM (transcripts per million) for normalization across samples.

Differential expression analysis was performed using DESeq2 [61]. Read counts were extracted using featureCounts [62] with -Q 20, --fracOverlap 0.8. and genes with |log2(Fold Change)| > 1 and adjusted *p*-value (Padj) ≤ 0.05 were considered differentially expressed. Multiple testing corrections were applied using the Benjamini–Hochberg method. RT-qPCR was employed to detect the gene expression level.

GO (Gene Ontology) and KEGG (Kyoto Encyclopedia of Genes and Genomes) pathway enrichment analyses were conducted on differentially expressed genes to identify biological processes and pathways involved, using zebrafish as the reference database for functional annotation.

### 4.5. Promoter Analysis of Ydsphk1

The candidate promoter sequences of the *Ydsphk1* were obtained from the assembled genome sequence (GeneBank: GCA_902410095.1) [63]. Six primer pairs were designed using Primer Blast (NCBI) to generate promoter deletion fragments of different lengths (Table 4). These fragments were cloned into the pGL3-basic vector, and the recombinant plasmids were confirmed by double digestion. Plasmids were co-transfected with the internal control plasmid pRL-TK into HEK293T cells using Lipo8000™ (Beyotime Biotechnology, Shanghai, China) transfection reagent. After 24 h, the luminescence intensities of firefly luciferase and Renilla luciferase were measured using a luminometer according to the Dual Luciferase Reporter Gene Assay Kit (Beyotime Biotechnology). The promoter activity was normalized by calculating the ratio of firefly to Renilla luciferase activity.

### 4.6. TFAP2A Binding Site Prediction

Transcription factor binding sites within the core promoter region of *Ydsphk1* were predicted using the FIMO (4.11.2) tool [64] within the MEME Suite and the JASPAR Vertebrate Transcription Factor Binding Site Database [65]. A matching *p*-value threshold of 1 × 10^−4^ was used to identify potential binding sites.

### 4.7. Regulatory Effect of TFAP2A on Ydsphk1 Promoter Activity

To assess the regulatory effect of TFAP2A on *Ydsphk1*, a recombinant vector PcDNA3.1-TFAP2A was constructed. This vector was co-transfected with *Ydsphk1* promoter deletion fragments and pRL-TK (internal control) into HEK293T cells in varying ratios (40:40:1). Dual luciferase activity was measured 24 h post-transfection to determine the effect of TFAP2A on promoter activity.

### 4.8. Real-Time qRT-PCR

Real-Time qRT-PCR primers for differentially expressed genes were designed using Primer Blast (NCBI) with *β-actin* (Table 3) from *Nibea albiflora* used as the internal control and with cDNA from experimental and control groups. Each group consisted of three biological replicates, with three technical replicates performed for each sample. The experiment was performed using a StepOne Plus Real-Time PCR System (Vazyme, Nanjing, China), following the instructions for the ChamQ Universal SYBR qPCR Master Mix (Vazyme, Nanjing, China). The reaction system (20 μL) consisted of: 10 μL of 2× ChamQ Universal SYBR qPCR Master Mix (Vazyme, Nanjing, China), 0.5 μL of forward and reverse primers each, 4 μL of cDNA template, and 5 μL of ddH_2_O. The thermal cycling conditions were as follows: 30 s at 95 °C for pre-denaturation, 10 s at 95 °C for denaturation, 30 s at 60 °C for annealing, and 30 s at 72 °C for extension, repeated for 40 cycles. A melt curve was performed at 95 °C for 15 s, 60 °C for 60 s, and 95 °C for 15 s. Three biological replicates were performed for each sample.

### 4.9. Statistical Analysis

All data were analyzed using SPSS 20.0 software (IBM, Armonk, NY, USA). Differences were considered statistically significant at *p* < 0.05, as determined by one-way ANOVA with LSD multiple comparisons.

## 5. Conclusions

This study establishes *Ydsphk1* as a pivotal regulator of the immune response in yellow drum (*Nibea albiflora*) against *Vibrio harveyi* infection. Overexpression of *Ydsphk1* significantly altered the expression of immune-related genes, notably upregulating *nsun5* and *hsp90aa1* while downregulating *Nfkbia* and *hmox1*, suggesting its role in modulating both inflammatory and stress response pathways. Promoter analysis identified two critical regulatory regions (−1931~−1679 bp and −419~+92 bp) and revealed that TFAP2A acts as a negative transcriptional regulator of *Ydsphk1*, further illuminating its transcriptional control mechanisms. Functional enrichment analyses highlighted the involvement of *Ydsphk1* in immune pathways, including NF-κB signaling and ferroptosis, emphasizing its multifaceted role in immune regulation and cellular stress responses during bacterial infections. These findings provide a comprehensive understanding of the molecular mechanisms underlying immune defense in yellow drum, offering valuable insights into strategies for improving disease resistance in aquaculture. Future research should focus on elucidating the broader regulatory networks involving *Ydsphk1* and its interactions with key immune pathways, particularly NF-κB signaling, to fully leverage its potential for enhancing pathogen resistance in aquaculture practices.

## Figures and Tables

**Table 1:** Summary of RNA-Seq data quality.

Group	RIN	Raw Data (Gb)	Clean Data (Gb)	Map Rate (%)
3-1	7.20	7.56	7.32	100.00%
3-2	10.00	6.84	6.59	100.00%
3-3	9.80	5.93	5.66	100.00%
Sphk1-1	10.00	7.27	6.99	100.00%
Sphk1-2	10.00	6.49	6.24	100.00%
Sphk1-3	10.00	7.60	7.25	100.00%

Note: groups 3-1, 3-2, and 3-3 are designated as controls for transferring *pcDN43.1(-) Myc-His B* plasmid; groups Sphk1-1, Sphk1-2, and Sphk1-3 are designated as Experimental for transferring *pcDN43.1(-) Myc-His B-YsSPHK1* plasmid. RIN: RNA integrity number. Raw data: Illumina raw sequencing data. Clean data: quality control and filtering from raw data by fastp. Map rate: genome alignment coverage for clean data.

**Table 2:** The fold change information and adjusted *p*-values of differentially expressed genes.

Gene ID	Group	log2(Fold Change)	−log10(Padj)
*Sphk1*	up	15.042	29.567
*Nsun5*		5.811	20.587
*Loxhd1*		3.919	10.389
*Kcnk10*		3.718	1.323
*Hamp*		2.710	1.827
*Vwde*		2.609	3.877
*Hsp90aa1*		2.484	3.877
*Faim2*		2.136	5.869
*Bag3*		1.569	3.422
*Dld*		1.557	1.448
*Tpcn1*		1.444	5.046
*Dnajb1*		1.440	1.340
*Dpys*		1.086	1.842
*Hmox*	down	−3.998	9.778
*Cmpk2*		−3.538	0.048
*Herc4*		−2.487	1.532
*Herc5*		−2.325	2.990
*Dcx*		−2.180	1.660
*Nfkbia*		−1.975	2.936
*Prdm1*		−1.837	1.794
*Tap2*		−1.822	1.323
*Kat8*		−1.424	1.320
*Gsto1*		−1.415	3.191
*Gnl*		−1.306	2.115
*Blvrb*		−1.279	1.519

Note: log2(Fold Change) represents the expression ratio of a gene between the experimental group and the control group in a logarithmic base-2 scale. Positive log2(Fold Change) indicates the genes is upregulated in the experimental group compared to the control group. Negative log2(Fold Change) means the gene is downregulated.

**Table 3:** Genes and gene-specific primers used for gene clone and RT-qPCR.

Gene Name	Primer Name	Primer Sequence (5′-3′)	PCR Product Length (bp)	Purpose
*SPHK1*	*pEGFP-SPHK1*-F	ctaccggactcagatCTCGAGATGGAGAAAGACGCATCTGAACC	1731	Subcellular localization
	*pEGFP-SPHK1*-R	gtaccgtcgactgcaGAATTCCTCCACATATAAGTCTGGCCAGTCC		
*SPHK1*	*PcDNA3.1-SPHK1*-F	aacgggccctctagaCTCGAGATGGAGAAAGACGCATCTGAACC	1730	Overexpression
	*PcDNA3.1-SPHK1*-R	tagtccagtgtggtgGAATTCGTCCACATATAAGTCTGGCCAGTCC		
*TFAP2A*	*pcDNA3.1-TFAP2A*-F	aacgggccctctagaCTCGAGATGTTAGTGCACAGTTTTTCCGC	1316	
	*pcDNA3.1-TFAP2A*-R	tagtccagtgtggtgGAATTCCTTTCTGTGCTTCTCGTCTTTGTC		
*β-ACTIN*	*q-β-actin*-F	TTATGAAGGCTATGCCCTGCC	107	RT-qPCR analysis
	*q-β-actin*-R	TGAAGGAGTAGCCACGCTCTGT		
*NSUN5*	*q-nsun5*-F	AGGACGAAGCAGGAGA	196	
	*q-nsun5*-R	TGAGATGAGCAGGGAAT		
*KCNK10*	*q-kcnk10*-F	GGCTATGACCCTAAGACA	177	
	*q-kcnk10*-R	CAGGTAGAGCACGACAAC		
*HAMP*	*q-hamp*-F	ACTCGTGCTCGCCTTTA	220	
	*q-hamp*-R	ACCGCAGCCTTTGTTC		
*HSP90A*	*q-hsp90a*-F	TCTGGACCCGTAACCC	222	
	*q-hsp90a*-R	TGAAGACCCTGCGAAC		
*FAIM2*	*q-faim2*-F	AGGACGACATACAGGC	213	
	*q-faim2*-R	TTACGGATGAAGACCC		
*DLD*	*q-dld*-F	ACAATGACGGCTATAAGT	239	
	*q-dld*-R	GTTCACCAGGTCCACA		
*DPYSL3*	*q-dpysl3*-F	ATGACAGCATAAAGCAGGAG	135	
	*q-dpysl3*-R	CCGCCAAGAAGGTAAAGA		
*LAT8*	*q-kat8*-F	GGGAGCAAGAAGTGTCG	110	
	*q-kat8*-R	GCCCTCCTGTTCATTCA		
*TAP2*	*q-tap2*-F	TCTGATTCCCGAGTGT	147	
	*q-tap2*-R	ATCGAGCTGGTAGTTGA		
*NFKBIA*	*q-nfkbia*-F	CTCGCCTCCATCGTGT	235	
	*q-nfkbia*-R	CGCCGTAGTTAAAGCAGT		
*HMOX*	*q-hmox*-F	AACGCCTCCCATCCAG	196	
	*q-hmox*-R	CGTGAGCGACCAACAG		

Note: Lowercase letters represent vector sequences, and enzyme restriction site of EcoR I (GAATTC) and Xho I (CTCGAG) are underlined. The first column indicates the names of the target genes, while the second column lists the corresponding primers used for their amplification. The primers named pEGFP-SPHK1 and PcDNA3.1-SPHK1 were used for gene cloning and vector recombination. The recombinant vectors were used for subcellular localization and overexpression, respectively. pcDNA3.1-TFAP2A was used for the regulation analysis of the *Ydsphk1*. The primers prefixed with q- are specifically designed for RT-qPCR.

**Table 4:** Primers for amplification of *Ydsphk1* promoter fragments of varying lengths.

Segment Name	Primer Name	Primer Sequences (5′-3′)	Length (bp)
	pGL3-SPHK1-R	atcgcagatctcgagCCCGGGTCGTTGAGCATGTCGGTGAA	
pGL3-SPHK1-P1	pGL3-SPHK1-F1	ctatcgataggtaccGAGCTCCGGCTCGTGTGTAACAAACT	2023
pGL3-SPHK1-P2	pGL3-SPHK1-F2	ctatcgataggtaccGAGCTCTACAGTCAAGTCAACCATCAGG	1769
pGL3-SPHK1-P3	pGL3-SPHK1-F3	ctatcgataggtaccGAGCTCTGAAGCCTGCCAGCCTCTA	1505
pGL3-SPHK1-P4	pGL3-SPHK1-F4	ctatcgataggtaccGAGCTCAACATTCTGCGTTCCACACTG	1156
pGL3-SPHK1-P5	pGL3-SPHK1-F5	ctatcgataggtaccGAGCTCGTGACACCACATTGTTCTACCA	794
pGL3-SPHK1-P6	pGL3-SPHK1-F6	ctatcgataggtaccGAGCTCAAGTCCGTGGTGAGTTCTTCT	511

Note: Lowercase letters represent vector sequences, and enzyme restriction sites of *Eco*R I (GAATTC) and *Xho* I (CTCGAG) are underlined. pGL3-SPHK1-P1~P6 represents promoter fragments of different lengths recombinant with the pGL3 vector.

## Data Availability

The data supporting the findings of this study are available within the article. Additional datasets generated or analyzed during the current study are available from the corresponding author upon reasonable request. RNA sequencing data have been deposited in NCBI Sequence Read Archive (SRA) under accession number PRJNA1196147.

## Supplementary Materials

The following supporting information can be downloaded at: https://www.mdpi.com/article/10.3390/ijms252413641/s1.
